# Cultural adaptation of the mental health first aid guidelines for depression in Brazil: a Delphi expert consensus study

**DOI:** 10.1186/s12888-023-04566-6

**Published:** 2023-01-27

**Authors:** Simone Scotti Requena, Thais Alves Assumpção, Carlos Henrique Mesquita Peres, Amanda Vidotto Cerqueira, Alexandre Andrade Loch, Wenging Li, Nicola J. Reavley

**Affiliations:** 1 Centre for Mental Health, Melbourne School of Population and Global Health, The University of Melbourne, Melbourne, Australia; 2 Laboratorio de Neurociencias (LIM 27), Instituto de Psiquiatria, Hospital das Clinicas HCFMUSP, Faculdade de Medicina, Universidade de São Paulo, São Paulo, Brazil; 3 Instituto Nacional de Biomarcadores em Neuropsiquiatria (InBion), Conselho Nacional de Desenvolvimento Cientifico e Tecnologico, São Paulo, Brazil

**Keywords:** Depression, Mental health first aid (MHFA), Cultural adaptation, Delphi study, Brazil

## Abstract

**Background:**

Depression is a significant contributor to disability in Brazil, with most Brazilians affected by depression receiving no treatment. As the community, including family and friends, plays a crucial role in providing support for someone with depression, it is important that evidence-based resources are available to support people who wish to help. The aim of this study was to culturally adapt the English-language mental health first aid guidelines for assisting a person with depression for the Brazilian culture.

**Methods:**

A Delphi expert consensus study was conducted, with two expert panels; health professionals (*n* = 29) and people with lived experience of depression (*n* = 28). One hundred and seventy-four statements from the English-language guidelines were translated into Brazilian Portuguese and administered as a survey. Participants were asked to rate statements based on how appropriate those statements were for the Brazilian culture and to suggest new statements if appropriate.

**Results:**

Data were collected over two survey rounds. Consensus was achieved on 143 statements. A total of 133 statements were adopted from the English-language guidelines, whereas 10 new endorsed statements were generated from suggestions of the two expert panels.

**Conclusions:**

There were similarities between the English-language and Brazilian guidelines, mainly related to family involvement and the value of empathy. More research on dissemination and incorporation of the guidelines into the Mental Health First Aid (MHFA) training course for Brazil is required.

**Supplementary Information:**

The online version contains supplementary material available at 10.1186/s12888-023-04566-6.

## Background

Depression is a major contributor to disability globally. Compared to 369 diseases and injuries, depressive disorders were the 13th leading cause of burden worldwide in 2019 [1]. According to the 2019 Global Burden of Disease study, almost 4% of the global population lives with depression, which is about 280 million people from all age groups [2]. In Brazil, almost nine million people were affected by depression in 2019 [2]. Even though depressive disorders are treatable, more than three-quarters of the Brazilian population does not receive any treatment for depression, with the treatment gap in the Northern region of Brazil reaching over 90% [3].

Mental health problems like depression are treated in the Brazilian public health care system mainly through community mental health services known as “Centers for Psychosocial Care (CAPS)”. CAPS were created during the Brazilian psychiatric reform process in 2001, when there was a major shift from a psychiatric hospital-based model of care, to a psychosocial model, centered around community-based mental health services [4]. However, the increase in community services since then has not been paralleled by a rise in public investment in mental health services [5], shown by a scarcity of community centers and the lack of resources where these centers exist [5, 6].

Beyond scarcity of resources and shortage of qualified mental health professionals, other reasons for the lack of treatment in low- and middle-income countries (LMICs) include poor mental health literacy and stigma toward people with mental health problems [7]. Evidence suggests that mental health literacy is a key predictor of mental health service use [8], particularly in LMICs [9, 10]. With regard to stigma, a study involving 1030 children and young people (8-21 years old) conducted in three different cities in Brazil found that stigma towards people with mental health problems was a major barrier for young people seeking help for mental health treatment [11]. Another study involving 46 Brazilian adults with mental health problems reported perceptions of Brazilian society as having difficulty empathizing, respecting, and understanding people’s suffering due to mental health problems. Study findings also pointed to the importance of considering family and close relationships in Brazil, as these may serve as sources of support as well as stigma and discrimination towards people with mental health problems [12]. This is in line with an evidence synthesis of 26 studies from Latin America and the Caribbean regions highlighting key culturally specific dimensions of stigma towards people with mental health problems, such as the importance of family, empathy, and close relationships [13]. Loch (2012) conducted a study in a public hospital in Brazil and found family stigma towards people with mental health problems to be a key predictor of hospital readmissions [14].

Because of the high prevalence of mental health problems such as depression, people in a person’s family and friendship networks are likely to come into contact with a person developing a mental health problem or in a mental health crisis. Evidence shows that assistance provided by family and friends can be essential sources of support, encouraging professional help-seeking, uptake of treatment, and effective self-help [15]. However, many people may lack the knowledge, confidence, or skills to help a person with a mental health problem. Therefore, the Mental Health First Aid (MHFA) training program was created to educate individuals from the community on how to support a person who is developing a mental health condition or is in a mental health crisis (e.g. at risk of suicide or having a traumatic experience) until professional help is received or the crisis ends [16]. MHFA training started in Australia and has expanded to more than 25 countries [17]. The training is grounded in the mental health first aid guidelines developed through Delphi consensus studies involving mental health professionals and individuals with lived experience [18–21]. A 2018 systematic review and meta-analysis (18 studies; 5936 participants) reported that the MHFA training is effective in reducing stigma, mental health literacy, and helping behavior up to 6 months after the training [22]. However, most of those studies were conducted in high-income countries, warranting cultural adaptions for LMICs.

Evidence suggests that the cross-cultural generalizability of the mental health first aid guidelines is likely, but there is still a need for cultural adaptation [23]. For instance, recent cultural adaptations of the mental health first aid guidelines for depression in China [24] and Sri Lanka [25] suggest that even though the adapted depression guidelines were similar to the English-language guidelines, some statements related to respect for one’s autonomy, involvement of the family [24, 25], and health communication style [25] were culturally-specific and needed to be added to the adapted guidelines. Likewise, the cultural adaptation of the mental health first aid guidelines for suicide in Brazil emphasized the importance of family and friends’ involvement when assisting someone at risk of suicide [26].

This study aimed to culturally adapt the English-language mental health first aid guidelines for assisting a person with depression to the Brazilian culture using the Delphi expert consensus method.

## Methods

This study used the Delphi method, which is a systematic way of gathering the insights of experts into a group consensus when experimental or epidemiological methods are not possible. The Delphi method involves several stages, during which a Delphi facilitator recruits the “expert” panels, compiles the surveys (a list of statements for the expert panels to rate), gathers and compares the responses of the expert panels, and converges responses across survey rounds using a pre-established statistical criterion to form consensus [27–29]. The expert panels in this study consisted of health professionals with experience in depression and people with lived experience of depression (including as carers). The panel members rated whether statements from the English-language guidelines were appropriate to be included in the Brazilian guidelines.

The current Delphi study involved four stages: (1) questionnaire development for the Round 1 survey, (2) identification and recruitment of two panels, (3) data collection and analysis over two survey rounds, and (4) guidelines development.

### Questionnaire development for the Round 1 survey

A senior psychiatrist (AAL) and three medical students (TAA, CHMP, AVC) translated the first round depression mental health first aid guidelines questionnaire from English to Portuguese language [30]. Minor changes to the translated statements were made to improve readability in the Portuguese language. The Round 1 survey included 174 statements organized in eight sections (see Table S1 in Additional file 1).

### Panel identification and recruitment

The expert panel consisted of two groups: (i) health professionals and (ii) consumers (people with lived experience) and carers. The inclusion criterion for the professional panel was to be a health practitioner working with people with depression, while the inclusion criteria for the consumers and carers panel was to have depression or be caring for someone with depression.

Participants were selected individually. For instance, for the health professional panel, individuals from universities and hospitals who were known to specialize in the treatment of depression, were contacted in person, by phone, or via email. This included individuals from the Institute of Psychiatry of the University of Sao Paulo. Individuals from the community and non-governmental organizations, such as outpatients from specialized services and social media support groups, were also contacted for the consumers and carers panel. Participants were briefly informed what the study was about, either verbally or by email, and then sent a survey link, which included more detailed information. Consent was obtained as a tick box before starting the survey. This study was approved by the University of Melbourne and the University of Sao Paulo ethics committees.

### Data collection and analysis

Eligible participants received a computer- and smartphone-friendly survey link hosted by Survey Monkey (https://pt.surveymonkey.com/). The first pages of the survey included information about the study, questions confirming eligibility for participation, and consent for participation. The next pages included socio-demographic questions about age, sex, the primary area of practice, and the setting of practice (for health professionals).

Participants were asked to rate how important they believed each statement was to be included in the Brazilian mental health first aid guidelines for helping someone with depression. All statements were rated on a five-point Likert scale (1 = Essential, 2 = Important, 3 = Depends/Don’t know, 4 = Not important, 5 = Should not be included). Using open-ended text boxes, participants were asked at the end of every survey section whether they would like to propose any new statements to be included in the guidelines. All comments were carefully reviewed by the research team and new statements were created and added to the Round 2 survey to be rated if these comments were new and actionable. A small amount of reimbursement for participants’ time was provided for those who completed both survey rounds (R$100 Brazilian reals; the equivalent of US$20).

Statements were immediately endorsed in the final guidelines if they were rated as essential or important by 80% or more of each panel at both rounds. If statements were rated as essential or important by 70 to 79% of each panel, or 80% or more of at least one panel and 70 to 79% from the other panel, they were re-rated at Round 2. Statements were immediately excluded at both rounds if they were rated by less than 70% by either panel. Cut-off values were based on previous Delphi studies of mental health first aid guidelines to help someone with depression [24, 25, 30].

The correlation between the statement ratings of the two panels was measured by Spearman’s correlation coefficient using R (version 1.4.1103).

### Guidelines development

Statements that were endorsed at both rounds – rated as essential or important by 80% or more of each panel – were assembled into eight sections. New endorsed statements without novel ideas were deleted, and vague statements were refined by two authors (SSR, NR) to improve clarity. For the final guidelines, the statement “The first aider should know about the local pathways to professional help, e.g. referral from a GP in order to see a specialist” from the English-language guidelines was reworded to “The first aider should know about the local pathways to professional help, e.g. referral from a GP in order to see a specialist, in case the person will be using the public health stream” since a referral from a GP is not a compulsory pathway to see a specialist in the private health stream in Brazil. The final list of statements in Portuguese language was checked and refined by two team members, who were native speakers (AAL, SSR), and the adapted Brazilian guidelines were generated (see Additional file 2).

## Results

### Expert panel information

A total of 57 expert panelists (29 health professionals, 28 consumers) completed the Round 1 survey. The socio-demographic characteristics of each expert panel are shown in Table 1. Most health professional panelists were female (72.4%) aged 19–62 years (Mean = 36.7, SD = 11.5). Similarly, most consumers were females (60.7%) aged 18–55 years (Mean = 32.3, SD = 10.9). Primarily, health professionals were psychologists (*n* = 9), followed by medical students (*n* = 4), nurses (*n* = 4), nurse technicians (*n* = 3), mental health researchers (*n* = 3), and psychiatrists (*n* = 2). The setting of the practice of health professionals included government hospitals (*n* = 10), educational institutions (*n* = 6), community mental health services and private practices (both at *n* = 5). Overall, 27 panelists participated in Round 2 (13 health professionals, 14 consumers and carers). The retention rates for both panels are shown in Table 2. The average amount of time participants took to complete their surveys was 46 minutes for the Round 1 survey and 20 minutes for the Round 2 survey.

**Table 1:** The socio-demographic characteristics of all participants

Variable	Health professionals		Consumers and carers	
	Frequency	Percentage	Frequency	Percentage
	(***n*** = 29)	(%)	(***n*** = 28)	(%)
**Sex**				
Female	21	72.4%	17	60.7%
Male	8	27.6%	11	39.3%
**Age (years)**				
Range	19	62	18	55
Mean ± SD	36.7	11.5	32.3	10.9
**Area of practice**				
Psychologists^a^	9	31.0%	NA	NA
MD students	4	13.8%	NA	NA
Nurses	4	13.8%	NA	NA
Nurse technicians	3	10.3%	NA	NA
MH researchers	3	10.3%	NA	NA
Psychiatrists^b^	2	6.9%	NA	NA
Others^c^	2	6.9%	NA	NA
Missing	1	3.4%	NA	NA
**Setting of practice**				
Government hospital	10	34.5%	NA	NA
Educational Institution	6	20.7%	NA	NA
Private practice	5	17.2%	NA	NA
Community MH service	5	17.2%	NA	NA
Other	1	3.4%	NA	NA
Missing	2	6.9%	NA	NA

*MD* Medical doctor, *MH* Mental health

^a^Includes one student

^b^Includes one intern

^c^Includes one aged care worker and one mental health worker

**Table 2:** Participation of Delphi panelists in each round by panel

	Panel of consumers and carers	Panel of health professionals	All
Round 1	28	29	57
Round 2	14 (50%)	13 (45%)	27 (47%)

### Rating of statements

**Fig. 1:**
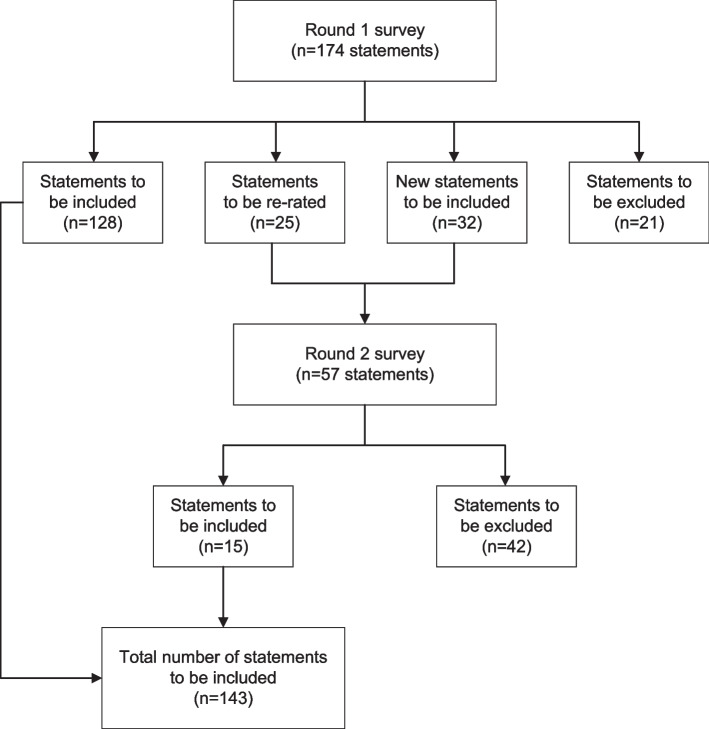
Overview of the study rounds

Consensus was reached on 143 statements for inclusion in the culturally adapted mental health first aid guidelines for Brazil after two survey rounds (see Additional file 1). The statements were divided into eight sections for helping a person with depression for Brazil: (1) How do I know if someone is experiencing depression?; (2) How should I approach someone who may be experiencing depression?; (3) How can I be supportive?; (4) Communicating effectively; (5) Difficulties the first aider may encounter; (6) Help-seeking; (7) What to do if the person doesn’t want help?; and (8) Concerns for safety. The number of statements included, excluded, and re-rated at both rounds is shown in Fig. 1.

A total of 174 statements were rated in the Round 1 survey, with 128 included, 25 re-rated, and 21 excluded (see Table S1 in Additional file 1). The Round 2 survey included 32 new statements created from panelists’ suggestions beyond the 25 statements that needed to be re-rated. Of these 57 statements, 15 statements were included and 42 were excluded (see Table S2 in Additional file 1). A total of 143 statements were included in the final adapted guidelines (see Table S3 in Additional file 1).

The consumer and carer panel had a higher average of ratings compared to the professional panel at both rounds (92% vs. 87% in round 1; 85% vs. 75% in round 2). There was a statistically significant relationship between the statements identified by the two panels, *r*_*s*_ = .59, *p* < .001). The correlation coefficient for round 2 was not calculated due to the unequal drop-out rates between panels.

### Differences between the English-language and Brazilian adapted guidelines

A total of 41 out of 174 (24%) statements from the English-language guidelines were excluded at rounds 1 (*n* = 21) and 2 (*n* = 20) (see Table S4 in Additional File 1 for the full list of statements from the English-language guidelines that were excluded from the Brazilian guidelines over two survey rounds). Additionally, 10 new statements were added to the Brazilian guidelines over six sections:

- Section 1: How do I know if someone is experiencing depression? (*n* = 2)
  1. The first aider should always listen when the person starts a conversation, even if the first aider does not know the person, empathy can help considerably.
  2. The first aider should listen to the advice from mental health professionals about how to deal with a person with depression.
- Section 2: How should I approach someone who may be experiencing depression? (*n* = 2)

3. The first aider should provide guidance on public mental health services, such as the closest “CAPS” to the person’s neighborhood.
4. The first aider should encourage the person to seek help from a “CAPS” or health center in the neighborhood.

- Section 3: How can I be supportive? (*n* = 2)

5. The first aider should check if there is someone in the family who can accompany the person when they seek treatment.
6. The first aider should not be influenced by the person’s feelings too much but show empathy towards the person.

- Section 4: Communicating effectively (*n* = 1)

7. The first aider should not use a mobile phone when talking to the person.

- Section 5: Difficulties the first aider may encounter (*n* = 3)

8. When offering help, the first aider should consider the person’s social class or culture and how this might impact on their help-seeking.
9. If the first aider and the person speak different languages, the first aider should look for someone to help with the language.
10. The first aider should respect the person’s cultural or religious beliefs about death and suicide.

## Discussion

This study aimed to culturally adapt the English-language mental health first aid guidelines for depression to the Brazilian culture. This adaptation was accomplished by a two-round Delphi study involving 29 health professionals with experience in depression and 28 consumers and carers with lived experience of depression in Brazil. There were similarities and differences between the English-language and the Brazilian guidelines.

### Comparison with the English-language guidelines

Of the 174 English-language statements presented to participants, 128 statements were endorsed in the Round 1 survey, and five statements were endorsed in the Round 2 survey, showing a high level of similarity (i.e., 76% agreement) between the English-language and the Brazilian adapted mental health first aid guidelines for depression. Key differences involved excluding 41 original English-language statements and the addition of 10 new statements. Statements related to respect for the autonomy of a person with depression, such as respect for personal feelings and experiences, were excluded from the adapted guidelines. For instance, the following statements from the English-language guidelines “The first aider should be open to any opportunity that presents itself to talk about their concerns with the person” and “The first aider should know that allowing the person to talk about how they are feeling can help them feel better, not worse” received low ratings by both panels. This may be because mental health problems are stigmatized in Brazil [11], even among mental health professionals [31], and talking openly about a mental health problem may be considered demeaning to a person with depression in Brazil.

Furthermore, statements related to the first aider prioritizing the person’s safety above all were seen in the Brazilian adapted guidelines. For example, statements from the English-language guidelines “The first aider should not assume that any signs or symptoms they have noticed means that the person is experiencing depression” and “The first aider should not assume that the person’s symptoms are due to depression” were not endorsed by either panel. This may be because involuntary psychiatric admissions are seen as an acceptable measure to protect individuals and society, with one in five patients in Brazil being involuntarily admitted to hospitals [32]. Moreover, this idea of prioritizing the safety of a person with a mental health problem and involuntarily admitting them to hospitals may be a potential source of shame and stigma towards the person and their family within the Brazilian society [12–14].

Statements related to the importance of family involvement when providing support to someone with depression were also added to the Brazilian guidelines. For instance, a statement created from participants’ comments (“The first aider should check if there is someone in the family who can accompany the person in their treatment”) was endorsed by all health professionals and all consumers and carers, showing the centrality of family within the Brazilian culture [13]. The focus on family seen in this study is in line with previously adapted mental health first aid guidelines for depression for other countries with collectivist cultures such as Sri Lanka [25] and China [24], where family involvement was also an essential factor in their adaptation of the guidelines.

Moreover, empathy-related statements were found to be culturally specific to the Brazilian guidelines. Firstly, statements from the English-language guidelines related to the importance of empathy were highly endorsed by both panels in round 1; for example, over 95% of participants in both panels believed the statement “The first aider should know that the key attitudes involved in non-judgmental listening are acceptance, genuineness and empathy” should be included. Likewise, two of the newly created and highly endorsed statements were related to empathy. This is in line with what Mascayano and colleagues highlighted as a fundamental aspect of Latin culture – the importance of showing empathy [13].

### Differences between panels

Differences in ratings between the health professional and consumers and carers greater than ±10% were observed. For instance, panels had opposing views on the extent to which people with depression should be responsible for their recovery – all consumers and carers endorsed the statement “The first aider should know that recovery, for the most part, must be led by the person”, while only 62% of professionals endorsed this statement. Likewise, consumers and carers thought it was more important for people with depression to use self-help strategies that worked for them in the past. More than three quarters of the consumers and carers endorsed the statement “The first aider should encourage the person to use self-help strategies that have helped the person in the past”, while only half of the professionals endorsed this statement. Finally, consumers and carers thought it was more important to seek advice from people who have recovered from depression than health professionals did – 80% of the consumers and carers endorsed the statement “The first aider should learn more about depression by seeking advice from people who have experienced and recovered from depression”, while only 69% of the professionals endorsed this statement. This may reflect the relatively less well-developed consumer advocacy movement in countries such as Brazil, as well as a lower emphasis on the agency of the person with depression [33, 34].

### Considerations for future use of the adapted guidelines

This study aimed to culturally adapt the English-language mental health first aid guidelines for assisting a person with depression to Brazilian culture by using opinions and views from health professionals and individuals with lived experience of depression in Brazil. As these guidelines’ adaptation showed some similarities and differences from the English-language guidelines, this provides further support for the importance of culturally adapting the guidelines.

The Brazilian adapted guidelines will be a stand-alone document and may also be used to guide the development of MHFA training. It is, however, important to note that the statements from these guidelines, which were assembled into sections, should not be taken individually as they may be more effective when used as a whole. The guidelines and the MHFA training may play a role in improving mental health literacy and reducing stigma in Brazil, although further research into development, implementation and effectiveness in Brazil is required to assess this.

### Strengths and limitations

A strength of this study was the systematic and evidence-based method used to adapt the English-language depression guidelines for Brazil. This adaptation allowed aspects of this culture, such as family involvement and empathy, to be incorporated into the adapted guidelines. Another strength of this study was the participant diversity, including, critically, people with their own lived experience and experience as carers. This ensured that widely varying views and opinions were gathered, which is fundamental in Delphi studies [29].

A limitation of this study was the low retention rate of participants in round 2, with the 50% retention rate not reaching a recommended 70% rate, possibly due to the time required to complete the questionnaires. However, the retention rates between both panels were similar, meaning that it is unlikely that one group of expert panelists had undue influence on the results. This drop-out rate may also impact on the generalizability of the findings.

## Conclusions

The English-language mental health first aid guidelines for assisting someone with depression were culturally adapted for Brazil using the Delphi expert consensus method. This study included health professionals with experience with depression and people with lived experience of depression. Despite a high level of similarity between the English-language and Brazilian adapted guidelines, differences related to the autonomy of a person with depression, prioritizing safety, family involvement when assisting someone with depression, and the value of empathy when helping a person with depression were seen.

The adapted guidelines can be used as a stand-alone resource by individuals from the community seeking to offer mental health first aid to those in their social networks who are developing depression. In addition, these guidelines may help combat stigma against people with depression and improve mental health literacy related to depression in Brazilian society. However, additional research on the dissemination and incorporation of these guidelines into the MHFA training materials for Brazil is still needed.

## Supplementary Information

**Additional file 1.** All survey statements in the mental health first aid guidelines for depression in Brazil.

**Additional file 2.** Mental Health First Aid Guidelines for Depression in Brazil (in Portuguese).

## Data Availability

The data supporting our findings is attached as the Additional file 1, which contains all the statements that were presented to panelists, including their endorsement rates.

## Abbreviations

LMICs: Low- and middle-income countries
MHFA: Mental Health First Aid
