# Correction: Cultural adaptation of the mental health first aid guidelines for depression in Brazil: a Delphi expert consensus study

**DOI:** 10.1186/s12888-023-04589-z

**Published:** 2023-02-08

**Authors:** Simone Scotti Requena, Thais Alves Assumpção, Carlos Henrique Mesquita Peres, Amanda Vidotto Cerqueira, Alexandre Andrade Loch, Wenjing Li, Nicola J. Reavley

**Affiliations:** 1 Centre for Mental Health, Melbourne School of Population and Global Health, The University of Melbourne, Melbourne, Australia; 2 Laboratorio de Neurociencias (LIM 27), Instituto de Psiquiatria, Hospital das Clinicas HCFMUSP, Faculdade de Medicina, Universidade de São Paulo, São Paulo, Brazil; 3 Instituto Nacional de Biomarcadores em Neuropsiquiatria (InBion), Conselho Nacional de Desenvolvimento Cientifco e Tecnologico, São Paulo, Brazil

**Correction: BMC Psychiatry 23, 76 (2023)**

**https://doi.org/10.1186/s12888-023-04566-6**

Following publication of the original article [1], the authors identified an error in the author name of Wenjing Li.

The incorrect author name is: Wenging Li

The correct author name is: Wenjing Li

The author group has been updated above and the original article [1] has been corrected.
