# Supplementary material for: Cultural adaptation of the mental health first aid guidelines for depression in Brazil: a Delphi expert consensus study
Source: BMC Psychiatry. 2023 Jan 27;23:76. doi: 10.1186/s12888-023-04566-6 (PMC9881332; doi:10.1186/s12888-023-04566-6)
Supplement: Supplementary file 2 — Additional file 2. Mental Health First Aid Guidelines for Depression in Brazil (in Portuguese). [file 12888_2023_4566_MOESM2_ESM.docx]

**DEPRESSÃO: MHFA DIRETRIZES**

**COMO POSSO SABER SE ALGUÉM ESTÁ PASSANDO POR DEPRESSÃO?**

Você não deve tentar diagnosticar a pessoa com depressão, pois apenas um(a) profissional treinado(a) pode fazer isso. Não ignore os sintomas que você notou ou suponha que estes vão simplesmente embora.

É importante que você aprenda sobre a depressão para que você possa reconhecer os sintomas da depressão e ajudar alguém que possa estar desenvolvendo depressão. Encontre informações sobre a depressão, como, suas causas, sintomas e tratamentos, e quais serviços estão disponíveis na sua área. Você pode fazer isso lendo ou ouvindo a experiência em depressão de outras pessoas.

**Fatores de risco que podem contribuir para o desenvolvimento da depressão:**

- Histórico familiar;
- Transtornos psiquiátricos correlatos;
- Estresse crônico;
- Ansiedade crônica;
- Disfunções hormonais;
- Dependência de álcool e drogas ilícitas;
- Traumas psicológicos;
- Doenças cardiovasculares, endocrinológicas, neurológicas, neoplasias entre outras;
- Conflitos conjugais;
- Mudança brusca de condições financeiras e desemprego.

*Fonte:* <https://www.gov.br/saude/pt-br/assuntos/saude-de-a-a-z/d/depressao-1>

**Sinais e sintomas de depressão que você pode observar.**

Embora a experiência de depressão possa variar em severidade, por exemplo, desde se sentir irritável até ter ideias suicidas, a seguir estão alguns sinais e sintomas comuns que você pode notar na pessoa. Você deve se preocupar se estes sinais persistirem ao longo do tempo e afetar o funcionando da saúde da pessoa:

- Humor depressivo: sensação de tristeza, autodesvalorização e sentimento de culpa. Acreditam que perderam, de forma irreversível, a capacidade de sentir prazer ou alegria. Tudo parece vazio, o mundo é visto sem cores, sem matizes de alegria. Muitos se mostram mais apáticos do que tristes, referindo “sentimento de falta de sentimento”. Julgam-se um peso para os familiares e amigos, invocam a morte como forma de alívio para si e familiares. Fazem avaliação negativa acerca de si mesmo, do mundo e do futuro. Percebem as dificuldades como intransponíveis, tendo o desejo de por fim a um estado penoso. Os pensamentos suicidas variam desde o desejo de estar morto até planos detalhados de se matar. Esses pensamentos devem ser sistematicamente investigados;
- Retardo motor, falta de energia, preguiça ou cansaço excessivo, lentificação do pensamento, falta de concentração, queixas de falta de memória, de vontade e de iniciativa;
- Insônia ou sonolência. A insônia geralmente é intermediária ou terminal. A sonolência está mais associada à depressão chamada Atípica;
- Apetite: geralmente diminuído, podendo ocorrer em algumas formas de depressão aumento do apetite, com maior interesse por carbohidratos e doces;
- Redução do interesse sexual;
- Dores e sintomas físicos difusos como mal estar, cansaço, queixas digestivas, dor no peito, taquicardia, sudorese.

*Fonte:* <https://www.gov.br/saude/pt-br/assuntos/saude-de-a-a-z/d/depressao-1>

Cada indivíduo é diferente e nem todo mundo que está passando por depressão mostrará os sinais ou sintomas típicos de depressão. Muitas pessoas que sofrem de depressão também podem ser afetadas por outros problemas de saúde mental, como por exemplo, ansiedade ou uso de drogas/dependência química.

É importante que você escute quando uma pessoa iniciar uma conversa, mesmo sem conhecer a pessoa, pois empatia ajuda consideravelmente. Também é importante que você escute profissionais de saúde mental sobre como lidar com uma pessoa com depressão.

**COMO DEVO ABORDAR ALGUÉM QUE PODE ESTAR SOFRENDO DE DEPRESSÃO?**

Pergunte a pessoa se ela quer conversar com você ou se ela prefere conversar com uma outra pessoa.

Tente passar um tempo com a pessoa e fale de forma gentil com ela sobre suas preocupações, como por exemplo, diga a pessoa que ela parece um pouco para baixo hoje.

É importante escolher um lugar privado onde ambos se sintam confortáveis. Se a pessoa disser que está se sentindo mal ou triste, você deve perguntar há quanto tempo ela está se sentindo assim.

Quando aproximar-se da pessoa, esteja preparado para lidar com todas as reações possíveis, como, alívio, indiferença, e/ou raiva. Lembre-se de que os pensamentos, sentimentos e crenças da pessoa representam sua própria realidade e você deve estar preparado(a) para aceitá-los, sem questionar.

Algumas pessoas que se recuperaram da depressão podem apresentar reincidência dos seus sintomas. No entanto, mesmo que a pessoa tenha tido um episódio prévio de depressão, não suponha que ela vai saber lidar com o episódio presente.

Você deve conhecer fontes de informações de boa qualidade. Se você fornecer informações sobre depressão à pessoa, é importante que você forneça a pessoa recursos que sejam precisos e apropriados para suas situação. Não sobrecarregue a pessoa com muita informação ou muitos recursos.

Oriente a pessoa sobre os serviços públicos de saúde mental, como o Centro de Atenção Psicossocial (CAPS) perto da sua moradia, incentivando-la a buscar ajuda no CAPS ou no seu posto de saúde.

**Como saber se as informações são confiáveis e precisas?**

Faça as seguintes perguntas para determinar se uma fonte de informação é confiável:

- O(A) autor(a) é conhecido(a) e respeitado(a) no campo da depressão ou doença mental?
- As informações são de uma instituição or departamento confiável (por exemplo, conhecido e respeitado na area clínica ou organização de saúde, departamento governamental, instituição filiada a uma universidade)?
- Qual é a idade da informação? Uma boa regra é procurar materiais publicados nos últimos dez anos.
- Os autores incluíram uma data? Se não, você não tem ideia de quão antiga a informação é.
- Procure sites que terminam com .gov.br (do governo, como do Ministério da Saúde), de universidades renomeadas, ou de uma organização conhecida internacionalmente (ex: Organização Mundial da Saúde, OMS).
- As informações contém erros de ortografia ou gramática ruim? Isto indica que o site pode não ser confiável.
- É um blog, postagem no Facebook ou site de autoria própria? Estes podem não ser confiáveis. Sempre verifique as informações obtidas dessas fontes.

*Fonte: adaptado de* [*https://mhfa.com.au/sites/default/files/depression_-_mhfa_guidelines_2020_1.pdf*](https://mhfa.com.au/sites/default/files/depression_-_mhfa_guidelines_2020_1.pdf)

**COMO POSSO AJUDAR?**

**Trate a pessoa com respeito**

A situação e necessidades de cada pessoa são únicas. É importante respeitar a autonomia da pessoa, ao mesmo tempo em que considerar até que ponto a pessoa é capaz de tomar decisões por si própria e se a pessoa está em risco de se machucar ou machucar aos outros. É importante também, manter em sigilo qualquer discussão que tenha com a pessoa, a não ser que você esteja preocupado que a pessoa possa se machucar ou machucar alguém.

**Ofereça apoio emocional e suporte de uma forma consistente**

Apesar de você não poder compreender exatamente o que a pessoa está sentindo, diga a ela que você se importa e quer ajudá-la. Diga a pessoa que ela é importante pra você, e que ela não está sozinha. De uma maneira sensível e acolhedora, fale pra pessoa sobre mudanças que você tenha percebido.

É mais importante que você se importe de verdade, do que dizer “a coisa certa”. Diga a pessoa que, apesar de sua experiência ser muito pessoal e dolorosa, ela não está sozinha. Muitas vezes, só de você conversar ou estar junto, faz com que a pessoa saiba que alguém se importa com ela.

Verifique se há alguém da família que pode acompanhar a pessoa no seu tratamento.

Não se deixe influenciar demasiadamente pelos sentimentos da pessoa, mas demonstre empatia.

A pessoa genuinamente precisa de suporte e compreensão adicional. É importante ser paciente, persistente e encorajador quando for ajudar alguém com depressão.

Pessoas com depressão podem ser dominadas por medos irracionais. Você deve oferecer bondade e atenção a pessoa, mesmo que isto não seja recíproco.

**Incentive a pessoa a falar com você**

Encoraje a pessoa a falar sobre seus pensamentos, sentimentos, sintomas e qualquer outro problema que ela esteja tendo. Você também pode perguntar à pessoa se algo aconteceu com ela recentemente que está contribuindo para ela sentir como ela se sente, mas não a pressione a falar, caso ela não queira.

Se a pessoa não tiver energia ou vontade de falar como ela se sente, não a pressione a falar sobre o assunto. Disponibilize-se para conversar quando a pessoa estiver pronta. Se a pessoa achar difícil discutir seus pensamentos e sentimentos de forma aberta, diga a ela que existem serviços disponíveis pra falar com alguém, como por exemplo, serviços telefônicos de aconselhamento.

**Seja um(a) bom(a) ouvinte**

Você deve ajudar uma pessoa com depressão ouvindo-la atentamente, sem expressar julgamento. As principais atitudes envolvidas na escuta sem julgamento são: aceitação, genuinidade e empatia. Adote uma atitude de aceitação para com a pessoa, como:

abstendo-se de todo e qualquer julgamento que tenha feito dela ou de suas circunstâncias, como achar que a pessoa está sendo preguiçosa (não expresse isso!). Deixe de lado quaisquer crenças e reações negativas e foque nas necessidades da pessoa que está recebendo ajuda.

Você pode demonstrar sinceridade à pessoa usando: linguagem corporal que corresponda à sua comunicação verbal (por exemplo: dizer à pessoa que aceita e respeita seus sentimentos enquanto mantém uma postura aberta e um contato visual apropriado). Demonstre empatia à pessoa mostrando que ela é verdadeiramente ouvida e compreendida, como por exemplo, dizendo a pessoa: “deve ser difícil passar pelo que você está passando”.

Use os seguintes recursos não verbais para reforçar a comunicação não julgadora:

- Preste atenção redobrada ao que a pessoa diz.
- Observe quanto espaço a pessoa se sente confortável e respeite isto.
- Mantenha contato visual em um nível com o qual a pessoa se sinta o mais confortável.
- Evite gestos distrativos, como por exemplo, ficar apertando uma caneta, encarar outras coisas ou bater pés ou mãos, pois isso pode ser interpretado como falta de interesse da sua parte.
- Esteja ciente da linguagem corporal da pessoa, na medida em que isso dá dicas de como ela está se sentindo ou quão confortável ela se sente em conversar com você.

Não interrompa a pessoa quando ela estiver falando, especialmente quando ela estiver compartilhando suas opiniões ou experiências. É importante ouvir atentamente a pessoa, mesmo se o que ela está dizendo não parecer verdade. Respeite os sentimentos, valores pessoais e experiências da pessoa, mesmo que sejam diferentes dos teus valores ou mesmo que você discorde deles.

Outras maneiras de ser um(a) bom(a) ouvinte:

- Faça perguntas que mostrem que você se importa de verdade e que você quer entender o que a pessoa está dizendo.
- Pausas e silêncios são ok, mesmo que desconfortáveis, já que a pessoa pode precisar de tempo para pensar ou encontrar as palavras certas.
- Verifique se você está entendendo o que está sendo dito, reafirmando o que a pessoa falou e resumindo os fatos e sentimentos ditos.
- Ouça não apenas o que a pessoa está dizendo mas também como ela diz, como por exemplo, o seu tom de voz.
- Se a pessoa tiver atitudes estigmatizantes em relação à problemas mentais, faça o possível para trabalhar a aceitação.
- Não use o celular enquanto estiver conversando com a pessoa.

**Tenha expectativas realistas**

Você deve aceitar a pessoa como ela é e não ter expectativas irreais dela. Saiba que atividades diárias, como limpar a casa, pagar as contas ou alimentar os cachorros, podem parecer muito desgastantes para a pessoa. No entanto, não pressione a pessoa pra fazer atividades que ela sente que são demais para ela, e reconheça que a pessoa não está mentindo, ou é preguiçosa, fraca ou egoísta.

**Reconheça os pontos fortes da pessoa**

Se a pessoa julgar-se de maneira muito dura, como por exemplo, dizendo que é fraca ou um fracasso, ajude a pessoa a lembrar dos seus pontos fortes e reconheça qualquer esforço que a pessoa esteja fazendo para melhorar. Diga à pessoa que ela não é fraca ou um fracasso porque ela tem depressão, pois pessoas fortes e capazes também podem ter depressão, diga a pessoa que você não a considera um ser humano inferior por ela ter depressão.

**Dê à pessoa esperança de recuperação**

Encoraje a pessoa dizendo-a que, embora ela não acredite agora, com tempo e tratamento, ela vai sentir-se melhor.

**Fornecendo suporte contínuo**

Seja claro(a) e consistente em quais tipos de apoio você pode ou não oferecer à pessoa, e não faça promessas que você não pode cumprir. Seja honesto(a) sobre as limitações de seu papel como socorrista, como por exemplo, que você não é um(a) conselheiro(a) profissional. Seja honesto(a) sobre qual ajuda você pode e está disposto(a) a oferecer. Pergunte à pessoa se o que você está fazendo está ajudando ela, e o que mais você poderia fazer para ajuda-la.

**O que não ajuda?**

A depressão é uma doença e não é culpa da pessoa que ela esteja com depressão. Não adianta apenas dizer à pessoa pra ficar bem ou “se recomponha” ou “anime-se”. Se isto fosse possível, a pessoa o faria. Não diga à pessoa “está tudo na sua cabeça”, ou que ela só precisa se ocupar mais ou sair mais de casa.

Não diminuia ou menospreze os sentimentos da pessoa ao tentar dizer algo positivo, como por exemplo, “você não parece tão mal pra mim”, já que isto pode parecer depreciativo ou desdenhoso, no entanto, deve ser evitado.

Escolha cuidadosamente suas palavras para evitar causar ofensa, como por exemplo, não use rótulos que a pessoa possa achar estigmatizantes, tal como “doente mental”. Além disso, não use palavras relacionadas a um potencial diagnóstico quando falando com a pessoa, como por exemplo, “você tem transtorno depressivo”.

Evite dizer a pessoa que não há nada que você possa fazer sobre a situação dela.

Não pegue no pé da pessoa para que ela faça o que normalmente faria, e evite confrontos, exceto se for pra prevenir a pessoa cometer atos perigosos ou nocivos. Não sugira que a pessoa use álcool ou outras drogas para se sentir melhor.

**E SE EU TIVER DIFICULDADES AO FALAR COM O PESSOA?**

Seja paciente, mesmo quando a pessoa não estiver se comunicando bem, for repetitiva ou falar mais devagar ou menos claramente do que o normal. Tente ser o mais solidário possível. Não seja crítico(a) ou expresse sua frustração com a pessoa por ela não conseguir se comunicar bem. Não seja hostil ou sarcástico(a) quando a pessoa estiver com dificuldades para responder, em vez disso, aceite as respostas como o melhor que a pessoa tem a oferecer neste momento.

Tente observar qualquer comportamento irritante ou desagradável como parte da doença e não leve isto como algo pessoal. Se a pessoa ficar brava durante a conversa, tente manter a calma, validar a raiva da pessoa, e não fazer suposições sobre a causa da raiva. No entanto, não aceite abuso ou comprometa a sua própria saúde mental quando for ajudar a pessoa.

Se a pessoa não quiser falar com você, sugira que ela converse com outra pessoa.

**Se as diferenças culturais estiverem interferindo na sua capacidade de ajudar a pessoa**, esteja disposto(a) a ajustar o seu comportamento verbal e não verbal, por exemplo, a pessoa pode estar confortável com um nível de contato visual diferente ou pode estar acostumada a mais espaço pessoal que você. Esteja ciente de que a pessoa pode ter especificidades quanto à procura por ajuda para seus problemas de saúde mental deviso ao seu contexto cultural ou crenças religiosas. Se você e a pessoa falarem idiomas diferentes, busque alguém para ajudar com o idioma. Respeite as crenças culturais ou religiosas da pessoa em relação à morte e ao suicídio. Caso disponível, fale com um serviço de saúde mental especializado que trabalhe com pessoas de diversos contextos culturais.

Considere a classe social e cultural da pessoa que você está ajudando, e pense como isto pode interferir na busca de ajuda dela.

**DEVO INCENTIVAR A PESSOA A PROCURAR AJUDA PROFISSIONAL?**

É importante que você consiga reconhecer quando encorajar a pessoa a procurar ajuda profissional. Não ache que a depressão da pessoa vai simplesmente passar, é importante tratar a depressão cedo para conseguir resultados melhores. Também é importante que você não minta ou dê desculpas pro comportamento da pessoa, pois isso pode atrasar a sua obtenção de assistência. Procure ajuda profissional imediata se a pessoa estiver tendo alucinações ou delírios.

É importante que você discuta as opções que a pessoa tem para encontrar ajuda profissional e deve encorajá-la a usar estas opções. Caso a pessoa não saiba onde conseguir ajuda, ajude-la a buscar ajuda profissional.

Você deve saber os recursos locais disponíveis para ajudar a pessoa, inclusive os serviços que estão disponíveis na região da pessoa. Você deve saber quais os caminhos para chegar à ajuda profissional, como por exemplo, a passagem por um clínico geral para ser encaminhado a um especialista, caso a pessoa use o sistema público de saúde.

Discuta os benefícios de buscar ajuda profissional e pergunte à pessoa se ela pensa que se beneficiaria disto. Fale sobre a procura por ajuda profissional de modo que normalize este ato, por exemplo: fale sobre isso como uma ação natural a se tomar; explique que problemas de saúde mental são comuns e tratáveis. Quando falar com a pessoa sobre a procura por ajuda profissional, evite rotular os comportamentos ou sentimentos da pessoa como sintomas da depressão.

A depressão muitas vezes não é reconhecida por profissionais da saúde e pode levar um tempo para ter um diagnóstico. Incentive a pessoa a não desistir de buscar ajuda profissional.

**E AS ESTRATÉGIAS DE AUTO-AJUDA?**

A capacidade e o desejo da pessoa em usar estratégias de auto-ajuda vão depender do seu interesse e da severidade da depressão. Você deve desencorajar a pessoa a usar álcool ou outras drogas para se sentir melhor.

Se a pessoa estiver interessada em estratégias de auto-ajuda, incentive-a a usar estratégias apropriadas.

**CUIDADOS PESSOAIS?**

Saiba que ajudar uma pessoa com depressão pode evocar uma resposta emocional inesperada em você. Se o comportamento da pessoa tiver um efeito negativo em você, reconheça seus próprios sentimentos e lide com eles separadamente. Se você se sentir chateado(a) ou desgastado(a) depois de ajudar a pessoa, ache alguém com quem você possa conversar sobre seus sentimentos, sem compartilhar os detalhes pessoais da pessoa que você estava ajudando.

Dicas para manter sua saúde mental em dia inclui: manter uma alimentação saúdavel, praticar atividades físicas, colocar o corpo em movimento de forma regular, priorizar o sono, ter momentos dedicados às pessoas queridas (como conviver com amigos e familiares), reservar um tempo para o esporte e lazer, fazer atividades que te deixem felizes, como passeios, encontros com amigos, cinema, ler bons livros, sair para dançar, entre outros, estar em contato com a natureza, procurar algo que lhe dê prazer, desenvolver a fé, auto-conhecimento, ajudar o próximo ou fazer trabalho voluntário. *fonte:* [*http://biblioteca.cofen.gov.br/dicas-saude-mental/*](http://biblioteca.cofen.gov.br/dicas-saude-mental/)*.*

**E SE A PESSOA NÃO QUISER AJUDA?**

É importante que você conheça as barreiras que leva a pessoa a não buscar tratamento para depressão, como por exemplo, ela sentir que ninguém pode ajudá-la. Descubra se existem razões específicas para a pessoa não querer procurar ajuda profissional (como por exemplo, preocupações com finanças, não ter um(a) médico(a) que ela goste ou estar preocupada que será enviada ao hospital), já que às vezes essas razões são baseadas em crenças equivocadas, e podem ser superadas com ajuda. Diga a pessoa que caso ela venha a mudar de ideia e queira buscar ajuda, ela pode contatar você.

**A não ser que haja risco de dano a pessoa ou aos outros,** respeite o direito da pessoa, se ela não estiver disposta a procurar ou aceitar ajuda. Respeite a vontade da pesoa e não a pressione a procurar ajuda profissional antes que ela esteja pronta. Não use mentiras, coerção ou ameaças para assegurar ajuda profissional.

**E SE HOUVER RISCO DA PESSOA SE MACHUCAR OU MACHUCAR AOS OUTROS?**

Se a pessoa estiver em risco de se ferir ou ferir aos outros, você deve considerar a segurança da pessoa e dos outros em primeiro lugar, e fazer algo a respeito. Envolva a pessoa nas decisões sobre quem mais deve saber sobre a situação de risco.

Existem diretrizes no idioma **português** **adaptadas a cultura brasileira** de primeiros socorros sobre como ajudar uma pessoa com **pensamentos suicidas**. Familiarize-se com estas diretrizes, que podem ser baixadas [documento em português dentro de “Additional file 2” na seção “Supplementary Information”] através do link: <https://link.springer.com/article/10.1186/s12888-022-04042-7#Sec19>.

**OBJETIVO DESTAS DIRETRIZES**

Estas diretrizes foram elaboradas para ajudar o público a prestar primeiros socorros a alguém que pode ter depressão. O papel do socorrista é ajudar a pessoa até que ajuda profissional seja encontrada ou a crise se resolva.

**DESENVOLVIMENTO DESTAS DIRETRIZES**

Estas diretrizes são baseadas nas opiniões de especialistas de pessoas com experiência vivida em depressão (consumidores e cuidadores) e profissionais da saúde mental (clínicos, pesquisadores e educadores) no Brasil. A metodologia usada neste estudo foi baseada no seguinte estudo: *Offering mental health first aid to a person with depression: a Delphi study to re-develop the guidelines published in 2008. BMC Psychology 2019; 7:37.*

**COMO USAR ESTAS DIRETRIZES**

Essas diretrizes são um conjunto geral de recomendações. Cada indivíduo é único e é importante adaptar o seu apoio às necessidades da pessoa. Portanto, essas recomendações podem não ser apropriadas para todas as pessoas.

Estas diretrizes foram desenvolvidas como parte de um conjunto de diretrizes sobre como melhor ajudar uma pessoa com problemas de saúde mental. Essas outras diretrizes **em inglês** podem ser baixadas a partir de: [mhfa.com.au/resources/mental-health-first-aid-guidelines](https://mhfa.com.au/mental-health-first-aid-guidelines).

Embora estas diretrizes sejam protegidas por direitos autorais, elas podem ser reproduzidas livremente sem fins lucrativos desde que seja citada. Por favor, cite estas diretrizes da seguinte forma: Mental Health First Aid Australia. Depressão: MHFA Diretrizes, Brasil; 2022.

Dúvidas devem ser enviadas para: [mhfa@mhfa.com.au](mailto:mhfa@mhfa.com.au).
